# Supplementary material for: Survival after surgery for spinal metastatic disease: a nationwide multiregistry cohort study
Source: BMJ Open. 2021 Nov 1;11(11):e049198. doi: 10.1136/bmjopen-2021-049198 (PMC8562515; doi:10.1136/bmjopen-2021-049198)
Supplement: Author's manuscript [file bmjopen-2021-049198.draft_revisions.pdf]

BMJ Open is committed to open peer review. As part of this commitment we make the peer review history of every article we publish publicly available.

When an article is published we post the peer reviewers' comments and the authors' responses online. We also post the versions of the paper that were used during peer review. These are the versions that the peer review comments apply to.

The versions of the paper that follow are the versions that were submitted during the peer review process. They are not the versions of record or the final published versions. They should not be cited or distributed as the published version of this manuscript.

BMJ Open is an open access journal and the full, final, typeset and author-corrected version of record of the manuscript is available on our site with no access controls, subscription charges or pay-per-view fees (http://bmjopen.bmj.com).

If you have any questions on BMJ Open's open peer review process please email info.bmjopen@bmj.com

BMJ Open

Survival after surgery for spinal metastatic disease: A nation-wide multi-registry cohort study

Journal:	*BMJ Open*
Manuscript ID	bmjopen-2021-049198
Article Type:	Original research
Date Submitted by the Author:	19-Jan-2021

Complete List of Authors:	Carrwik, Christian; Uppsala University, Department of Surgical Sciences<br>Olerud, Claes; Uppsala University, Department of Surgical Sciences<br>Robinson, Yohan; Uppsala University, Department of Surgical Sciences; Armed Forces Centre for Defence Medicine, Department of Research and Development

Keywords:	Spine < ORTHOPAEDIC & TRAUMA SURGERY, ONCOLOGY, Neurosurgery < SURGERY

SCHOLARONE™
Manuscripts

I, the Submitting Author has the right to grant and does grant on behalf of all authors of the Work (as defined in the below author licence), an exclusive licence and/or a non-exclusive licence for contributions from authors who are: i) UK Crown employees; ii) where BMJ has agreed a CC-BY licence shall apply, and/or iii) in accordance with the terms applicable for US Federal Government officers or employees acting as part of their official duties; on a worldwide, perpetual, irrevocable, royalty-free basis to BMJ Publishing Group Ltd ("BMJ") its licensees and where the relevant Journal is co-owned by BMJ to the co-owners of the Journal, to publish the Work in this journal and any other BMJ products and to exploit all rights, as set out in our licence.

The Submitting Author accepts and understands that any supply made under these terms is made by BMJ to the Submitting Author unless you are acting as an employee on behalf of your employer or a postgraduate student of an affiliated institution which is paying any applicable article publishing charge ("APC") for Open Access articles. Where the Submitting Author wishes to make the Work available on an Open Access basis (and intends to pay the relevant APC), the terms of reuse of such Open Access shall be governed by a Creative Commons licence – details of these licences and which Creative Commons licence will apply to this Work are set out in our licence referred to above.

Other than as permitted in any relevant BMJ Author's Self Archiving Policies, I confirm this Work has not been accepted for publication elsewhere, is not being considered for publication elsewhere and does not duplicate material already published. I confirm all authors consent to publication of this Work and authorise the granting of this licence.

For peer review only

Title: Survival after surgery for spinal metastatic disease: A nation-wide multi-registry cohort study

Authors: Christian Carrwik MD (corresponding author), Claes Olerud MD PhD, Yohan Robinson MD PhD MBA

Christian Carrwik MD (corresponding author)

christian.carrwik@surgsci.uu.se

Department of Surgical Sciences

Uppsala University

SE-751 85 Uppsala

Sweden

Phone +46-(0)18-611 00 00

Claes Olerud MD PhD

claes.olerud@surgsci.uu.se

Department of Surgical Sciences

Uppsala University

Yohan Robinson MD PhD MBA
yohan.robinson@gu.se

1. Department of Surgical Sciences
   Uppsala University
Sweden
2. Department of Research and Development
Armed Forces Centre for Defence Medicine
Gothenburg
Sweden
3. Institute of Clinical Sciences
Sahlgrenska academy, Gothenburg University
Gothenburg
Sweden

For peer review only - http://bmjopen.bmj.com/site/about/guidelines.xhtml

Abstract

Objectives: To evaluate survival after surgery and indications for surgery due to spinal metastatic disease.

Design: A retrospective longitudinal multi-registry nationwide cohort study.

Setting: 19 public hospitals in Sweden with spine surgery service, where six university hospitals account for over 90 percent of the cases.

Participants: 1820 patients 18 years or older undergoing surgery due to spinal metastatic disease 2006-2016 and registered in Swespine, the Swedish national spine surgery register.

Interventions: Decompressive and/or stabilizing spine surgery due to spinal metastatic disease.

Primary outcome: Survival (median and mean) after surgery.

Secondary outcomes: Indications for surgery, types of surgery and causes of death.

Results: The median estimated survival after surgery was 6.2 months (95% CI 5.6-6.8) and the mean estimated survival time was 12.2 months (95% CI 11.4-13.1). Neurologic deficit was the most common indication for surgery and posterior decompression and stabilization was performed in 70.5% of the cases. A neoplasm was stated as the main cause of death for 97% of the patients.

Conclusion: Both median and mean survival times were well above the generally accepted thresholds for surgical treatment for spinal metastases, suggesting that patient selection for surgical treatment on a national level is adequate. Further research on quality of life after surgery and prognostication is needed.

Strengths and limitations of this study

For peer review only

For peer review only - http://bmjopen.bmj.com/site/about/guidelines.xhtml

- Large nation-wide study with reliable data on demographic and survival
- Includes data on indications, surgical methods and tumour types
- Multi-register study with linkage to cause of death register
- High level of missing data on quality of life before and after surgery
- No control group with patients treated only non-surgically

Key words

Tumour, spine, metastases, cancer, surgery, survival

Word count: 3724

Introduction

While there is strong evidence that surgical treatment can improve quality of life for patients with metastatic spine disease, predicting outcome after surgery is still a matter of extensive research.[1] The patients with spinal metastases often present with acute symptoms such as progressive neurological impairment, giving limited time to evaluation and decision making. While the surgery might have a positive effect on health-related quality of life (HRQoL), it is not intended to increase survival and is associated with complications.[2]

On the one hand, patients selected for surgery for spinal metastatic disease should have enough expected survival time to justify the risks with surgery. On the other hand, no patient should be excluded from potentially beneficial surgery. This dilemma highlights the need for tools for decision making in oncological spine surgery, and there is an abundance of scoring systems, developed to predict survival after surgery. Several commonly used scoring systems have problems estimating survival, which might withhold patients from potentially beneficial surgery.[3-5]

In recent years, mortality rates in several common types of cancer known to cause metastatic spine disease has decreased. This is especially evident in high-income countries, where a higher level of

For peer review only - http://bmjopen.bmj.com/site/about/guidelines.xhtml

For peer review only

access to spine surgery could be expected.[6] A drawback of most scoring systems is that they often are based on retrospective studies of patients treated decades ago, not reflecting recent advancements in oncology.[7]

The main objective of this study was to study survival after surgery for metastatic spine disease stratified by tumour type in a nationwide cohort. Secondary objects of investigation were indications for surgery, types of surgery and causes of death.

Materials & methods

Study design

This is an observational multi-registry cohort study of adult patients undergoing surgery due to metastatic spine disease. The study protocol follows RECORD guidelines[8] and is approved by the Regional Ethical Review Board in Uppsala (no. 2012/133).

Setting

All patients were treated in Sweden, a developed country with a national universal healthcare system. [9] There is virtually no private cancer care available, thus the public healthcare institutions cover most of the included cases. The patients were treated at 19 different public hospitals, where six university hospitals accounted for 1646 (90%) of the cases.

Since reporting of healthcare quality is a legal requirement, most Swedish hospitals choose to report using the national healthcare quality registries. Swespine is the Swedish register for spine surgery and has been in use since 1993. The module for spinal metastatic disease was introduced 2006 and the register as whole has a coverage (reflecting the spine surgery centres connected to the register) of about 90% and a completeness (number of procedures entered in the register) of 75% during the period.[10]

For peer review only

For deceased patients, the date of death and cause of death are registered in the death certificate by the examining physician, or the pathologist when a post-mortem is performed. The certificates are entered into the Cause of Death register, where date of death and cause of death (CoD) are part of a death record. The register contains data from 1961 and onwards. The physician responsible for the patient is requested to report the cause of death to the register. If no CoD is reported, a reminder is eventually sent out. After reminders 1-2% of the deaths still lack a reason of death and are then coded as "Death NOS", R99.9 according to International Statistical Classification of Diseases version ten (ICD-10).

As every individual residing in Sweden has a personal identification number (PIN), linkage of registries using the PIN is possible.

Participants

Patients 18 years and older, undergoing surgery August 1st 2006-August 25th 2018 due to spinal metastatic disease and registered in the spinal metastasis module of Swespine, were included. (Figure 1)

Patient and Public Involvement

Patients or the public were not involved in the design, or conduct, or reporting, or dissemination plans of our research.

Variables

Survival

Survival after surgery was the primary endpoint and was calculated using the time difference from the date of surgery as reported in Swespine and the date of death from the CoD register. Surviving patients until the date of registry extraction were treated as censored cases. For the whole cohort

For peer review only - http://bmjopen.bmj.com/site/about/guidelines.xhtml

(including patients alive as of December 31st 2016), the survival was estimated using the Kaplan Meier method.

To stratify survival per tumour type, we used data on tumour type from the CoD register rather than from Swespine to compensate for the high level of missing data on tumour type in Swespine.

Type of tumour

The type of primary tumour is reported by the surgeon in Swespine as known or unknown at the time of surgery. If known, the surgeon can choose one of eight types to specify. The available choices are prostate, breast, renal, thyroid, lung, hematologic, gastrointestinal or other.

Cause of death

The CoD Register includes the CoD diagnosis according to ICD-10. If the reported main cause of death was a neoplasm (ICD-10 codes C00-D48), this was considered as the primary tumour in the analysis. In cases where multiple reasons of death were reported, the first neoplasm diagnosis (ICD-10 codes C00-D48) among the reported codes was used.

The quality of the Swedish CoD register is described as high with a completeness of 97%. While the autopsy rates have dropped during in the last decades, the quality of the reporting was not necessarily negatively affected thanks to better diagnostic tools.[11] A retrospective study on 5675 patients with prostate cancer published in 2009 reported a 86% correlation between the registered CoD and the CoD according to the reviewed records.[12]

Indications for surgery

In Swespine, the surgeon reports the indication for surgery. The possible indications are neurologic deficits, pain, progressing deformity or any combination of those.

For peer review only - http://bmjopen.bmj.com/site/about/guidelines.xhtml

For peer review only

Neurologic function

The patient's neurologic function is reported according to the Frankel scale A-E, where A means a complete neurologic deficit and E means full motoric and sensory function.[13]

Types of surgery

The surgeon reports the type of decompressive surgery (posterior/anterior and levels) and types of implants used (yes/no to posterior and anterior implants) to Swespine. If both questions regarding implants were answered "no", we assumed that the patient was operated with decompression only.

In Swespine, the spinal levels of surgery are reported by the surgeon. The levels of decompression and stabilisation (if applicable) are reported, as well as the most proximal level of tumour mass addressed by the procedure. The most proximal level of tumour mass is used in this study to determine if the tumour mass is on cervical, thoracic or lumbosacral level. The decompression and/or stabilisation might cover more proximal levels than the reported level.

Quality of life

The patients are requested to assess their HRQoL before surgery by filling in an EQ-5D form. EQ-5D measures HRQoL in five dimensions and is widely used and validated in several medical fields.[14] Six weeks after surgery, the patient is requested to fill in another form and the values before and after surgery are reported to Swespine.

Data Sources / measurement

The spinal metastasis registry extract was provided by Swespine and sent to the Swedish National Board of Health and Welfare, where dates and causes of death were linked with the spinal metastasis registry dataset. An anonymised dataset was provided to the authors while the key remained with the agency.

For peer review only - http://bmjopen.bmj.com/site/about/guidelines.xhtml

Bias

All patients in this cohort were selected to undergo surgery and had probably better functional status than patients not selected for surgery.

Registration of patient data in quality registers in Sweden is regulated by law and it is assumed that the patient agrees to participation, unless otherwise stated.[15] This reduces the risk of selection bias.

For peer review only

Study size

The study size was determined by the number of patients with spinal metastatic disease registered in Swespine. No power calculations were made prior to the study.

Quantitative variables

Type of surgery

The surgeon-reported data on surgery includes type of surgery (anterior and/or posterior decompression. type of implant used) and spinal levels of surgery. The most proximal part of tumour mass addressed by the surgery is registered and this value is used to classify the metastases as cervical, thoracic or lumbosacral.

Statistical methods

All calculations were made with the statistical software IBM SPSS Statistics version 26, including the survival estimations. The survival analysis was with the Kaplan Meier method. To test the statistical significance in survival between groups, the Breslow test was used.

Data access and cleaning methods

The authors have full access to the Swespine database, and the anonymised dataset with the

patients in the spinal metastases cohort. This dataset matches the dataset received from the CoD register and no further cleaning of cases has been made.

Linkage

The authors were provided with an already linked dataset. The linkage was performed by the National Board of Health and Welfare.

Results

Participants

1820 adult patients were included in the analysis. The patients were treated at 19 different hospitals in Sweden. Six university hospitals accounted for 1646 (90%) of the cases.

Descriptive data

The included patients were 18 to 95 years old at the time of surgery (mean age 67 years, 67% male).

Outcome data

Survival

The estimated median survival after surgery was 6.2 months (95% CI 5.6-6.8) and the mean survival 12.2 months (95% CI 11.4-13.1). As of January 31st 2016, 531 of the patients were alive and 1289 were deceased. 49.0 percent were dead within six months after surgery and 68.6 percent were dead within twelve months after surgery. (Figure 2)

For peer review only

For peer review only - http://bmjopen.bmj.com/site/about/guidelines.xhtml

Cause of death

1253 of the deceased patients (97%) had a tumour diagnosis (ICD 10 codes C00-D48) listed as the main cause of death, according to the CoD register. In 26 of the cases (2.0%) no neoplasm diagnosis was reported as a contributing cause of death.

Survival per tumour type

In 773 cases (45.8%), the tumour type was known before surgery according to Swespine, while it was stated as unknown in 301 cases (17.9%). There was missing data regarding 612 cases (36.3%). (Table 1)

The median estimated survival after surgery for patients with known primary tumour was 6.3 months and mean survival 12.0 months. For unknown primary tumour, median survival was estimated to 5.3 months and mean survival 14.6 months. This difference was not statistically significant (p=0.68). (Table 2)

To compensate for the high level of missing data on tumour type in Swespine, we stratified survival per tumour type with linked data from the CoD register. This part of the analysis included data on the 1289 patients deceased per December 31st 2016. This lowered the level of missing data on tumour type to 3 percent. However, as only deceased patients could be included, the expected survival time could be underestimated. Of the three sub-analysed tumour groups, patients with breast cancer had the longest estimated median and mean survival. (Table 3, Figure 3)

Indications for surgery

The most common indication for surgery was neurologic deficit, either alone or in combination with other symptoms. Data on indication was missing in 41 percent of the cases (Table 4).

For peer review only - http://bmjopen.bmj.com/site/about/guidelines.xhtml

Neurologic function

The neurologic status before surgery according to the Frankel scale is reported to Swespine. Frankel level C was the most commonly reported level while data was missing in 43% of the cases. (Table 5)

Types of surgery

Posterior decompression was performed in 1592 cases while 141 patients were decompressed anteriorly. 63 patients (3.5%) were decompressed both anteriorly and posteriorly. Data regarding decompression was missing in 1.3 percent of the cases. It is unclear from the data if this means that no decompression was done. (Table 6)

Types of implants

The most common surgical procedure in this material was decompression combined with stabilisation. Posterior implants were used almost seven times more frequently than anterior implants. In 378 cases no implants were used at all. It is unclear whether data missing indicates that no implants were used or not. The type of implant (model/manufacturer) is reported to Swespine and the most common type used in this cohort was a posterior pedicle screw system.

Spinal levels of surgery

Thoracic lesions were far more common than lumbosacral and cervical lesions. Th4 is the most common proximal level addressed by surgery in this material. (Figure 4)

Quality of life

1180 of the patients (64.8%) had a registered value for EQ-5D as assessed prior to surgery and 911 patients (50.1%) had HRQoL data assessed six weeks after surgery. The concordance of the two groups was low, as only 411 (22.6%) had data registered both before and after surgery.

For peer review only - http://bmjopen.bmj.com/site/about/guidelines.xhtml

Discussion

Key results

To the best of our knowledge, this is one of the largest single-country cohorts with prospective baseline data, reliable survival data and recently treated patients. The Swedish system with a unique PIN makes it possible to cross-reference and obtain data from several registries, which is one of the strengths with this study.

The median survival after surgery for metastatic spine disease in this cohort is by margin above the levels recommended in predictive scoring systems where there is a time-based level for expected survival (usually three months), indicating that more patients could be eligible for surgery given the relatively long survival.[16] As previously shown by our group, the use of old prediction models may withhold patients from surgery as they tend to underestimate survival, and this study shows once again the need for reliable prediction tools.[17]

The Global Spine Tumour Study Group (GSTSG) has published several articles based on an international cohort of comparable size. While the patients in that cohort show higher survival rate one year after surgery, the mean age is lower (60 years versus 67 years in our material) and the distribution of cancer types is different. Patients with breast cancer and lung cancer were both more common than prostate cancer in the GSTSG cohort and sarcomas were included as well. The heterogeneity between these cohorts complicates any comparative analysis.[3, 18]

Not surprisingly, almost every patient with spinal metastatic disease in our cohort died of cancer rather than of other reasons. This emphasises the need to minimise complications after surgery in this fragile group.

The focus of this study is on survival after surgery, which is an important factor when making treatment decisions. However, we recognise that the main goal of the treatment is not to prolong

For peer review only - http://bmjopen.bmj.com/site/about/guidelines.xhtml

the life but rather to increase the HRQoL for the patient. Unfortunately, the amount of missing data regarding HRQoL in the spinal metastasis module of Swespine is very high. Almost eight out of ten patients have missing data on HRQoL and based on this data we cannot make any conclusions on the effects of the surgery regarding HRQoL. The explanation for the high level of missing data is probably multi-factoral.

The distribution of the metastases in the spine, where the thoracic spine accounts for the majority followed by the lumbar and cervical spines, is in line with other recent findings.[19, 20]

Limitations

The main limitation of this study is the lack of a non-surgical control group and the lack of reliable reporting of adverse events. Swespine has a system for reporting adverse advents in connection to the surgery, but finding late complications is a cumbersome task including manual assessment of multiple medical records and out of the scope for this study. Others authors have concluded that the rate of adverse events after spine surgery probably is under-reported and we have no reason to believe that the situation is different in Sweden.[21]

Interpretation

Survival after surgery for spinal metastatic disease in this cohort s well above the recommended minimum and surgery might be considered in even more cases. Further research should emphasise on prediction of survival and evaluation of HRQoL.

Generalisability

The results of this study do not apply to all patients with spinal metastatic disease, given the heterogeneity between different cancer forms and the lack of a non-surgical control group.

For peer review only - http://bmjopen.bmj.com/site/about/guidelines.xhtml

Footnotes

Author Contributions

CC drafted the manuscript and made the statistical analyses. CO was involved in the study design and reviewed the manuscript. YR designed the study, wrote the ethical board application and reviewed the manuscript.

Funding

This research received no specific grant from any funding agency in the public, commercial or not-for-profit sectors.

Competing interests

Dr. Carrwik and Dr. Robinson have nothing to disclose. Dr. Olerud reports personal fees from Johnson & Johnson (paid speaker on course) outside the submitted work.

Ethics approval

This study was approved by the Regional Ethical Review Board in Uppsala (reference 2012/133).

Data availability statement

Source data was accessed from Swespine and The National Cause of Death Register. Swespine is available to researchers subject to application (http://www.swespine.se) and the National Cause of Death Register is open-access, see https://www.socialstyrelsen.se/en/statistics-and-data/registers/

For peer review only

For peer review only - http://bmjopen.bmj.com/site/about/guidelines.xhtml

Tables

**Type of primary tumor**	**n**	**%**
Known	773	47.5
Unknown	301	18.5
Data missing	612	37.6
**Total**	**1626**	**100**

Table 1. Primary tumor as known before surgery reported to Swespine.

**Primary tumor**	**Cases**	**Median estimated survival after surgery. months (95% CI)**	**Mean estimated survival after surgery. months (95% CI)**
Known (n=773)	773	6.3 (5.4-7.2)	12.0 (10.9-13.2)
Unknown (n=301)	301	5.3 (3.8-6.8)	14.6 (12.0-17.2)

Table 2. Estimated survival after surgery for patients with known versus unknown primary tumor at the time of surgery, as reported to Swespine.

Primary tumor	Cases	Median estimated survival after surgery. months (95% CI)	Mean estimated survival after surgery. months (95% CI)
Prostate	405	7.2 (6.2-8.2)	11.5 (10.2-12.8)
Breast	111	10.4 (5.2-15.6)	17.7 (14.6-20.9)
Lung	163	3.4 (2.4-4.4)	7.6 (6.0-9.3)

Table 3. Survival per tumor type, as reported in the CoD register.

**Indication for surgery**	**n**	**%**
Neurologic deficit (A)	684	38
Pain (B)	161	9
Progressive deformity (C)	19	1
A+B	154	9
A+C	11	0.6
B+C	20	1
A+B+C	31	2
Data missing	740	41
**Total**	**1820**	**100**

Table 4. Indications for surgery as reported to Swespine.

**Frankel level**	**n**	**%**
A	37	2.0
B	78	4.3
C	430	23.6
D	298	16.4
E	192	10.5
Missing data	785	43.1

For peer review only

**Total**	**1820**	**100**

Table 5. Neurologic status before surgery.

**Type of decompression**	**n**	**%**
Posterior	1592	87.5
Anterior	141	7.7
Posterior and anterior	63	3.5
Data missing	24	1.3
**Total**	**1820**	**100**

Table 6. Types of decompression as reported to Swespine.

Figure Legends

Figure 1. Flowchart for inclusion and exclusion in the study.

Figure 2. Survival after surgery, all tumour types.

Figure 3. Survival after surgery per primary tumour.

Figure 4. Number of cases per proximal level addressed by surgery.

For peer review only - http://bmjopen.bmj.com/site/about/guidelines.xhtml

For peer review only

References

1. Patchell RA, Tibbs PA, Regine WF, et al. Direct decompressive surgical resection in the treatment of spinal cord compression caused by metastatic cancer: a randomised trial. The Lancet. 2005;366(9486):643-8.
2. Dea N, Versteeg A, Fisher C, et al. Adverse events in emergency oncological spine surgery: a prospective analysis. Journal of neurosurgery Spine. 2014:1-6.
3. Choi D, Ricciardi F, Arts M, et al. Prediction Accuracy of Common Prognostic Scoring Systems for Metastatic Spine Disease: Results of a Prospective International Multicentre Study of 1469 Patients. Spine (Phila Pa 1976). 2018;43(23):1678-84.
4. Choi D, Fox Z, Albert T, et al. Prediction of Quality of Life and Survival After Surgery for Symptomatic Spinal Metastases: A Multicenter Cohort Study to Determine Suitability for Surgical Treatment. Neurosurgery. 2015;77(5):698-708; discussion
5. Kim J, Lee S-H, Park S-J, et al. Analysis of the predictive role and new proposal for surgical strategies based on the modified Tomita and Tokuhashi scoring systems for spinal metastasis. World journal of surgical oncology. 2014;12:245.
6. Torre LA, Siegel RL, Ward EM, Jemal A. Global Cancer Incidence and Mortality Rates and Trends--An Update. Cancer epidemiology, biomarkers & prevention : a publication of the American Association for Cancer Research, cosponsored by the American Society of Preventive Oncology. 2016;25(1):16-27.
7. Wang M, Bünger CE, Li H, et al. Predictive Value of Tokuhashi Scoring Systems in Spinal Metastases, Focusing on Various Primary Tumor Groups: Evaluation of 448 Patients in the Aarhus Spinal Metastases Database. Spine. 2012;37(7):573-82.
8. Benchimol EI, Smeeth L, Guttmann A, et al. The REporting of studies Conducted using Observational Routinely-collected health Data (RECORD) Statement. PLoS Med. 2015;12(10):e1001885.
9. Anell A, Glenngard AH, Merkur S. Sweden health system review. Health systems in transition. 2012;14(5):1-159.
10. Stromqvist B, Fritzell P, Hagg O, Jonsson B, Sanden B. Swespine: the Swedish spine register : the 2012 report. Eur Spine J. 2013;22(4):953-74.
11. Brooke HL, Talback M, Hornblad J, et al. The Swedish cause of death register. European journal of epidemiology. 2017;32(9):765-73.
12. Fall K, Stromberg F, Rosell J, Andren O, Varenhorst E. Reliability of death certificates in prostate cancer patients. Scandinavian journal of urology and nephrology. 2008;42(4):352-7.
13. Frankel HL, Hancock DO, Hyslop G, et al. The value of postural reduction in the initial management of closed injuries of the spine with paraplegia and tetraplegia. I. Paraplegia. 1969;7(3):179-92.
14. Rabin R, de Charro F. EQ-5D: a measure of health status from the EuroQol Group. Annals of medicine. 2001;33(5):337-43.
15. Levay C. Policies to foster quality improvement registries: lessons from the Swedish case. Journal of internal medicine. 2016;279(2):160-72.
16. Tokuhashi Y, Matsuzaki H, Oda H, Oshima M, Ryu J. A revised scoring system for preoperative evaluation of metastatic spine tumor prognosis. Spine (Phila Pa 1976). 2005;30(19):2186-91.
17. Carrwik C, Olerud C, Robinson Y. Predictive Scores Underestimate Survival of Patients with Metastatic Spine Disease: A Retrospective Study of 315 Patients in Sweden. Spine (Phila Pa 1976). 2019.

For peer review only - http://bmjopen.bmj.com/site/about/guidelines.xhtml

18. Depreitere B, Ricciardi F, Arts M, et al. How good are the outcomes of instrumented debulking operations for symptomatic spinal metastases and how long do they stand? A subgroup analysis in the global spine tumor study group database. Acta neurochirurgica. 2020.
19. Amelot A, Terrier LM, Cristini J, et al. Approaching spinal metastases spread profile. Surgical oncology. 2019;31:61-6.
20. Onken JS, Fekonja LS, Wehowsky R, Hubertus V, Vajkoczy P. Metastatic dissemination patterns of different primary tumors to the spine and other bones. Clinical & experimental metastasis. 2019;36(6):493-8.
21. Dea N, Versteeg A, Fisher C, et al. Adverse events in emergency oncological spine surgery: a prospective analysis. Journal of neurosurgery Spine. 2014;21(5):698-703.

For peer review only

For peer review only - http://bmjopen.bmj.com/site/about/guidelines.xhtml

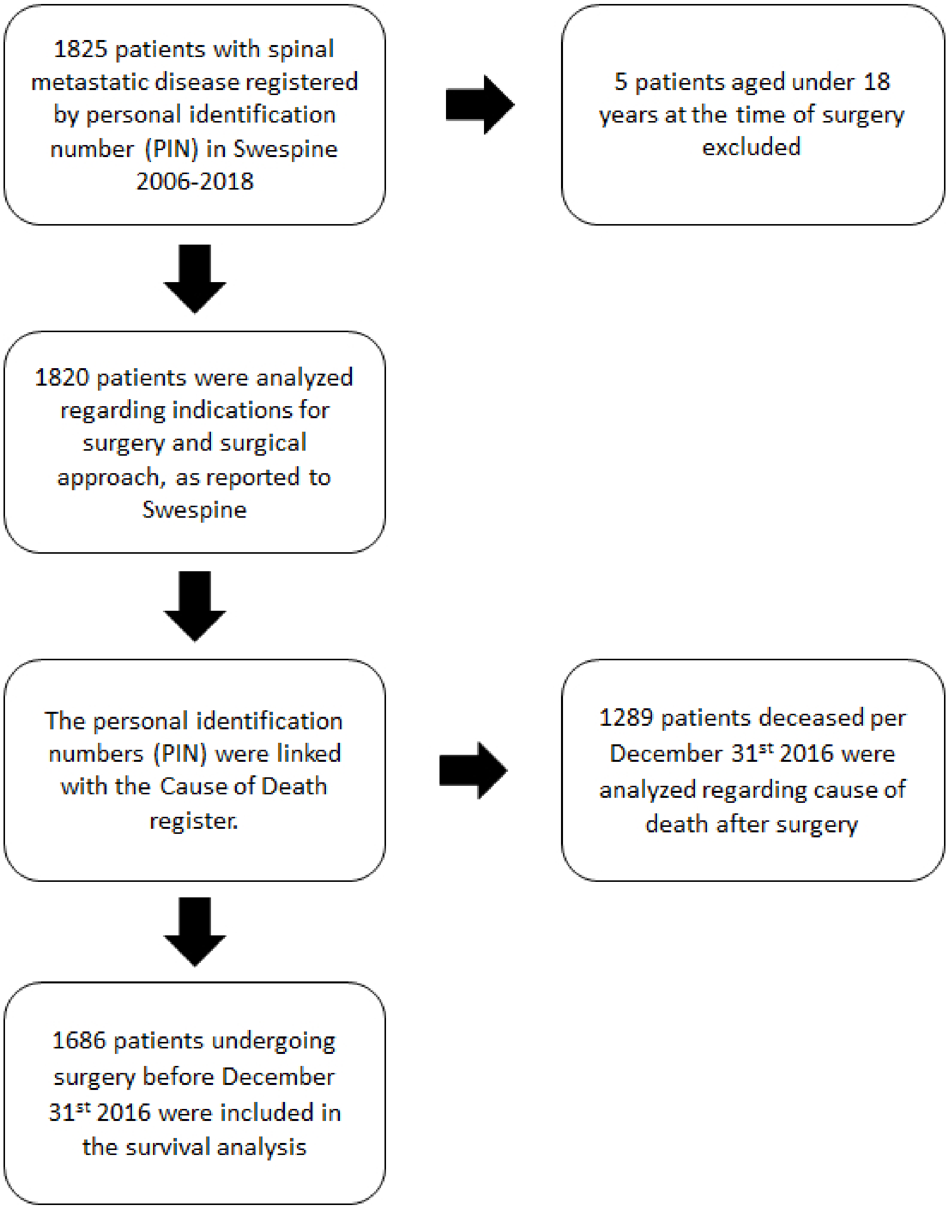

Flowchart for inclusion and exclusion in the study.

116x147mm (120 x 120 DPI)

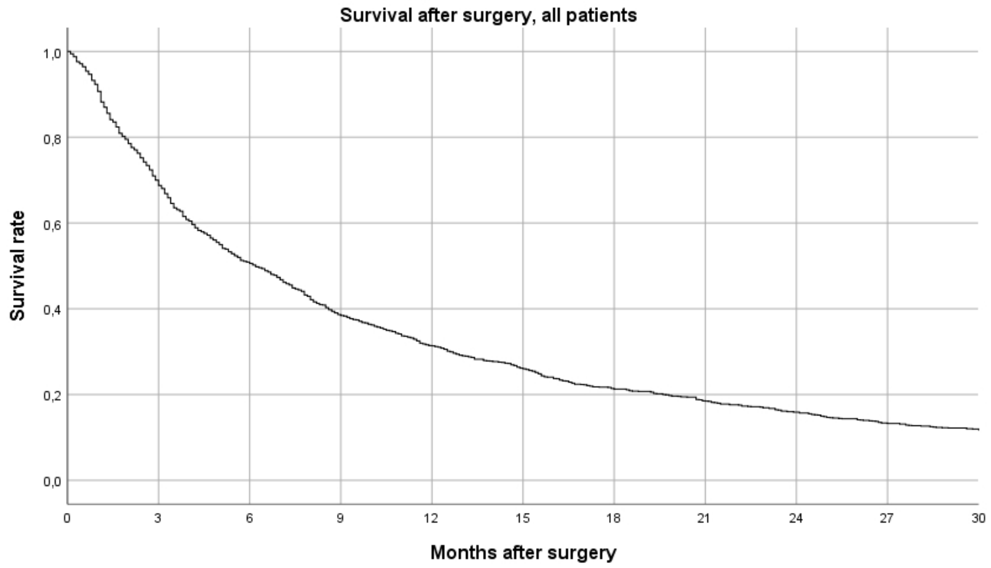

Survival after surgery, all tumour types.

179x107mm (120 x 120 DPI)

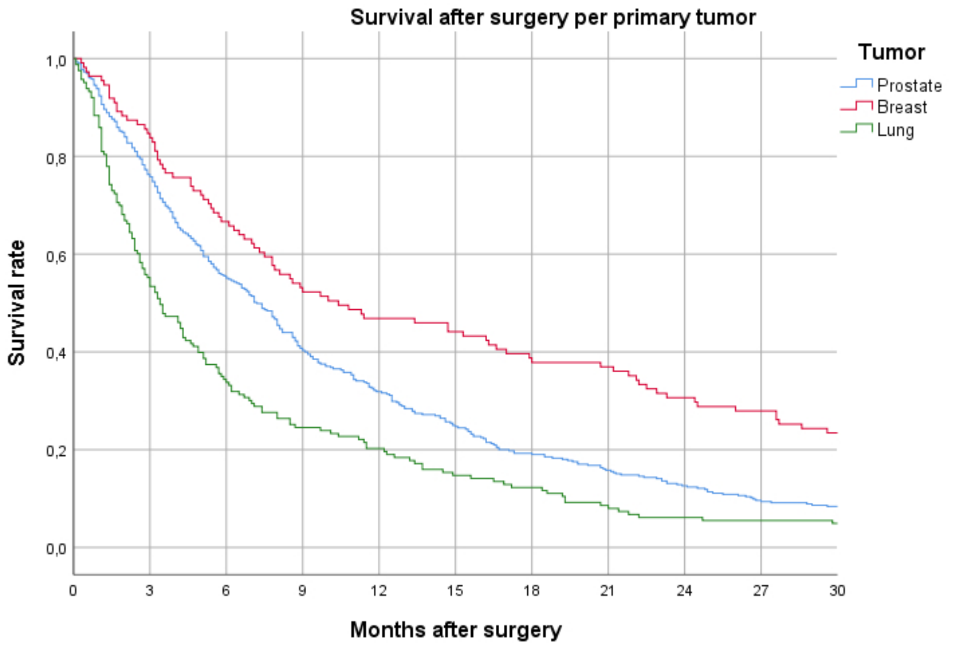

Survival after surgery per primary tumour.

157x108mm (120 x 120 DPI)

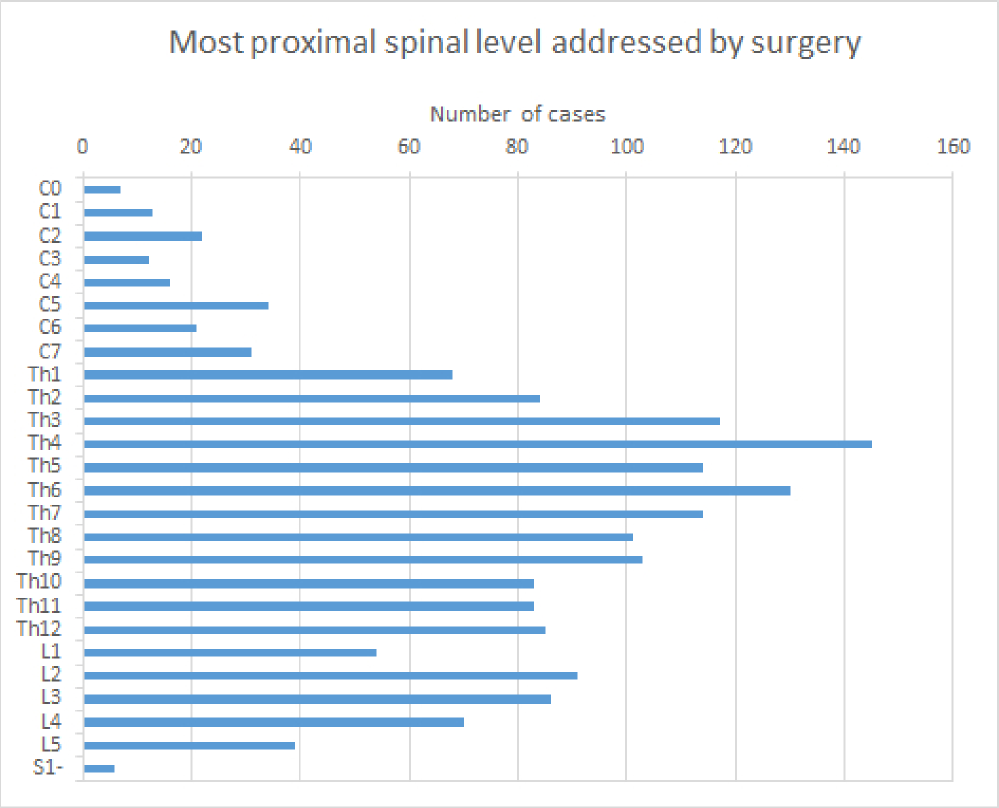

Number of cases per proximal level addressed by surgery.

111x90mm (120 x 120 DPI)

Reporting checklist for cohort study.

Based on the STROBE cohort guidelines.

Instructions to authors

Complete this checklist by entering the page numbers from your manuscript where readers will find each of the items listed below.

Your article may not currently address all the items on the checklist. Please modify your text to include the missing information. If you are certain that an item does not apply, please write "n/a" and provide a short explanation.

Upload your completed checklist as an extra file when you submit to a journal.

In your methods section, say that you used the STROBE cohortreporting guidelines, and cite them as:

von Elm E, Altman DG, Egger M, Pocock SJ, Gotzsche PC, Vandenbroucke JP. The Strengthening the Reporting of Observational Studies in Epidemiology (STROBE) Statement: guidelines for reporting observational studies.

For peer review only

		Reporting Item	Page Number
**Title and abstract**			
Title	#1a	Indicate the study’s design with a commonly used term in the title or the abstract	1

Abstract	#1b	Provide in the abstract an informative and balanced summary of what was done and what was found	2
**Introduction**			
Background / rationale	#2	Explain the scientific background and rationale for the investigation being reported	3
Objectives	#3	State specific objectives, including any prespecified hypotheses	2
**Methods**			
Study design	#4	Present key elements of study design early in the paper	2
Setting	#5	Describe the setting, locations, and relevant dates, including periods of recruitment, exposure, follow-up, and data collection	2
Eligibility criteria	#6a	Give the eligibility criteria, and the sources and methods of selection of participants. Describe methods of follow-up.	2
Eligibility criteria	#6b	For matched studies, give matching criteria and number of exposed and unexposed	5
Variables	#7	Clearly define all outcomes, exposures, predictors, potential confounders, and effect modifiers. Give diagnostic criteria, if applicable	5
Data sources / measurement	#8	For each variable of interest give sources of data and details of methods of assessment (measurement). Describe comparability of assessment methods if there is more than	7

For peer review only

		one group. Give information separately for for exposed and unexposed groups if applicable.	
Bias	#9	Describe any efforts to address potential sources of bias	7
Study size	#10	Explain how the study size was arrived at	7
Quantitative variables	#11	Explain how quantitative variables were handled in the analyses. If applicable, describe which groupings were chosen, and why	7
Statistical methods	#12a	Describe all statistical methods, including those used to control for confounding	
7			
Statistical methods	#12b	Describe any methods used to examine subgroups and interactions	7
Statistical methods	#12c	Explain how missing data were addressed	7
Statistical methods	#12d	If applicable, explain how loss to follow-up was addressed	7
Statistical methods	#12e	Describe any sensitivity analyses	
7			
**Results**			
Participants	#13a	Report numbers of individuals at each stage of study—eg numbers potentially eligible, examined for eligibility,	8

For peer review only

		confirmed eligible, included in the study, completing follow-up, and analysed. Give information separately for for exposed and unexposed groups if applicable.	
Participants	#13b	Give reasons for non-participation at each stage	8
Participants	#13c	Consider use of a flow diagram	
8			
Descriptive data	#14a	Give characteristics of study participants (eg demographic, clinical, social) and information on exposures and potential confounders. Give information separately for exposed and unexposed groups if applicable.	8
Descriptive data	#14b	Indicate number of participants with missing data for each variable of interest	
8			
Descriptive data	#14c	Summarise follow-up time (eg, average and total amount)	
9			
Outcome data	#15	Report numbers of outcome events or summary measures over time. Give information separately for exposed and unexposed groups if applicable.	
9			
Main results	#16a	Give unadjusted estimates and, if applicable, confounder-adjusted estimates and their precision (eg, 95% confidence	8

For peer review only

		interval). Make clear which confounders were adjusted for and why they were included	
Main results	#16b	Report category boundaries when continuous variables were categorized	8
Main results	#16c	If relevant, consider translating estimates of relative risk into absolute risk for a meaningful time period	
8			
Other analyses	#17	Report other analyses done—eg analyses of subgroups and interactions, and sensitivity analyses	8
**Discussion**			
Key results	#18	Summarise key results with reference to study objectives	11
Limitations	#19	Discuss limitations of the study, taking into account sources of potential bias or imprecision. Discuss both direction and magnitude of any potential bias.	11
Interpretation	#20	Give a cautious overall interpretation considering objectives, limitations, multiplicity of analyses, results from similar studies, and other relevant evidence.	12
Generalisability	#21	Discuss the generalisability (external validity) of the study results	13
**Other Information**			

For peer review only

Funding	#22	Give the source of funding and the role of the funders for the present study and, if applicable, for the original study on which the present article is based	13

The STROBE checklist is distributed under the terms of the Creative Commons Attribution License CC-BY. This checklist was completed on 18. January 2021 using https://www.goodreports.org/, a tool made by the EQUATOR Network in collaboration with Penelope.ai

For peer review only

Survival after surgery for spinal metastatic disease: A nation-wide multi-registry cohort study

Journal:	*BMJ Open*
Manuscript ID	bmjopen-2021-049198.R1
Article Type:	Original research
Date Submitted by the Author:	29-Sep-2021

Complete List of Authors:	Carrwik, Christian; Uppsala University, Department of Surgical Sciences<br>Olerud, Claes; Uppsala University, Department of Surgical Sciences<br>Robinson, Yohan; Uppsala University, Department of Surgical Sciences; Armed Forces Centre for Defence Medicine, Department of Research and Development

**Primary Subject Heading**:	Surgery
Secondary Subject Heading:	Oncology
Keywords:	Spine < ORTHOPAEDIC & TRAUMA SURGERY, ONCOLOGY, Neurosurgery < SURGERY

I, the Submitting Author has the right to grant and does grant on behalf of all authors of the Work (as defined in the below author licence), an exclusive licence and/or a non-exclusive licence for contributions from authors who are: i) UK Crown employees; ii) where BMJ has agreed a CC-BY licence shall apply, and/or iii) in accordance with the terms applicable for US Federal Government officers or employees acting as part of their official duties; on a worldwide, perpetual, irrevocable, royalty-free basis to BMJ Publishing Group Ltd ("BMJ") its licensees and where the relevant Journal is co-owned by BMJ to the co-owners of the Journal, to publish the Work in this journal and any other BMJ products and to exploit all rights, as set out in our licence.

The Submitting Author accepts and understands that any supply made under these terms is made by BMJ to the Submitting Author unless you are acting as an employee on behalf of your employer or a postgraduate student of an affiliated institution which is paying any applicable article publishing charge ("APC") for Open Access articles. Where the Submitting Author wishes to make the Work available on an Open Access basis (and intends to pay the relevant APC), the terms of reuse of such Open Access shall be governed by a Creative Commons licence – details of these licences and which Creative Commons licence will apply to this Work are set out in our licence referred to above.

Other than as permitted in any relevant BMJ Author's Self Archiving Policies, I confirm this Work has not been accepted for publication elsewhere, is not being considered for publication elsewhere and does not duplicate material already published. I confirm all authors consent to publication of this Work and authorise the granting of this licence.

For peer review only

Title: Survival after surgery for spinal metastatic disease: A nation-wide multi-registry cohort study

Authors: Christian Carrwik MD (corresponding author), Claes Olerud MD PhD, Yohan Robinson MD PhD MBA

Christian Carrwik MD (corresponding author)

christian.carrwik@surgsci.uu.se

Department of Surgical Sciences

Uppsala University

SE-751 85 Uppsala

Sweden

Phone +46-(0)18-611 00 00

Claes Olerud MD PhD

claes.olerud@surgsci.uu.se

Department of Surgical Sciences

Uppsala University

Yohan Robinson MD PhD MBA
yohan.robinson@gu.se

1. Department of Surgical Sciences
   Uppsala University

Sweden

2. Armed Forces Centre for Defence Medicine

Gothenburg
Sweden

3. Institute of Clinical Sciences

Sahlgrenska academy, Gothenburg University
Gothenburg
Sweden

For peer review only - http://bmjopen.bmj.com/site/about/guidelines.xhtml

Abstract

Objectives: To evaluate survival after surgery and indications for surgery due to spinal metastatic disease.

Design: A retrospective longitudinal multi-registry nationwide cohort study.

Setting: 19 public hospitals in Sweden with spine surgery service, where six university hospitals account for over 90 percent of the cases.

Participants: 1820 patients 18 years or older undergoing surgery due to spinal metastatic disease 2006-2016 and registered in Swespine, the Swedish national spine surgery register.

Interventions: Decompressive and/or stabilizing spine surgery due to spinal metastatic disease.

Primary outcome: Survival (median and mean) after surgery.

Secondary outcomes: Indications for surgery, types of surgery and causes of death.

Results: The median estimated survival after surgery was 6.2 months (95% CI 5.6-6.8) and the mean estimated survival time was 12.2 months (95% CI 11.4-13.1). Neurologic deficit was the most common indication for surgery and posterior stabilization was performed in 70.5% of the cases. A neoplasm was stated as the main cause of death for 97% of the patients.

Conclusion: Both median and mean survival times were well above the generally accepted thresholds for surgical treatment for spinal metastases, suggesting that patient selection for surgical treatment on a national level is adequate. Further research on quality of life after surgery and prognostication is needed.

Strengths and limitations of this study

For peer review only - http://bmjopen.bmj.com/site/about/guidelines.xhtml

- Large nation-wide study with reliable data on demographic and survival
- Includes data on indications, surgical methods, and tumour types
- Multi-register study with linkage to cause of death register
- High level of missing data on quality of life before and after surgery
- No control group with patients treated only non-surgically

Key words

Tumour, spine, metastases, cancer, surgery, survival

Word count: 3459

Introduction

While there is strong evidence that surgical treatment can improve quality of life for patients with metastatic spine disease, predicting outcome after surgery is still a matter of extensive research.[1] The patients with spinal metastases often present with acute symptoms such as progressive neurological impairment, giving limited time to evaluation and decision making. While the surgery might have a positive effect on health-related quality of life (HRQoL), it is not intended to increase survival and is associated with complications.[2]

On the one hand, patients selected for surgery for spinal metastatic disease should have enough expected survival time to justify the risks with surgery. On the other hand, no patient should be excluded from potentially beneficial surgery. This dilemma highlights the need for tools for decision making in oncological spine surgery, and there is an abundance of scoring systems, developed to predict survival after surgery. Several commonly used scoring systems have problems estimating survival, which might withhold patients from potentially beneficial surgery.[3-5]

For peer review only

For peer review only - http://bmjopen.bmj.com/site/about/guidelines.xhtml

In recent years, mortality rates in several common types of cancer known to cause metastatic spine disease has decreased. This is especially evident in high-income countries, where a higher level of access to spine surgery could be expected.[6] A drawback of most scoring systems is that they often are based on retrospective studies of patients treated decades ago, not reflecting recent advancements in oncology.[7]

The main objective of this study was to study survival after surgery for metastatic spine disease stratified by tumour type in a nationwide cohort. Secondary objects of investigation were indications for surgery, types of surgery and causes of death.

Materials & methods

Study design

This is an observational multi-registry cohort study of adult patients undergoing surgery due to metastatic spine disease. The study protocol follows RECORD guidelines[8] and is approved by the Regional Ethical Review Board in Uppsala (no. 2012/133).

Setting

All patients were treated in Sweden, a developed country with a national universal healthcare system. [9] There is virtually no private cancer care available, thus the public healthcare institutions cover most of the included cases. The patients were treated at 19 different public hospitals, where six university hospitals accounted for 1646 (90%) of the cases.

Since reporting of healthcare quality is a legal requirement, most Swedish hospitals choose to report using the national healthcare quality registries. Swespine is the Swedish register for spine surgery and has been in use since 1993. The module for spinal metastatic disease was introduced 2006 and the register as whole has a coverage (reflecting the spine surgery centres connected to the register)

For peer review only - http://bmjopen.bmj.com/site/about/guidelines.xhtml

of about 90% and a completeness (number of procedures entered in the register) of 75% during the period.[10]

For deceased patients, the date of death and cause of death are registered in the death certificate by the examining physician, or the pathologist when a post-mortem is performed. The certificates are entered into the Cause of Death register, where date of death and cause of death (CoD) are part of a death record. The register contains data from 1961 and onwards. The physician responsible for the patient is requested to report the cause of death to the register. If no CoD is reported, a reminder is eventually sent out. After reminders 1-2% of the deaths still lack a reason of death and are then coded as “Death NOS”, R99.9 according to International Statistical Classification of Diseases version ten (ICD-10).

As every individual residing in Sweden has a personal identification number (PIN), linkage of registries using the PIN is possible.

Participants

Patients 18 years and older, undergoing surgery August 1st 2006-August 25th 2018 due to spinal metastatic disease and registered in the spinal metastasis module of Swespine, were included. (Figure 1)

Patient and Public Involvement

Patients or the public were not involved in the design, or conduct, or reporting, or dissemination plans of our research.

For peer review only

For peer review only - http://bmjopen.bmj.com/site/about/guidelines.xhtml

Variables

Survival

Survival after surgery was the primary endpoint and was calculated using the time difference from the date of surgery as reported in Swespine and the date of death from the CoD register. Surviving patients until the date of registry extraction were treated as censored cases. For the whole cohort (including patients alive as of December 31st, 2016), the survival was estimated using the Kaplan Meier method.

Due to the high level of missing data on tumour type in Swespine, we stratified data on tumour type from the CoD register and from Swespine separately, for comparison and validation of register data.

Type of tumour

The type of primary tumour is reported by the surgeon in Swespine as known or unknown at the time of surgery. If known, the surgeon can choose one of eight types to specify. The available choices are prostate, breast, renal, thyroid, lung, hematologic, gastrointestinal or other.

Cause of death

The CoD Register includes the CoD diagnosis according to ICD-10. If the reported main cause of death was a neoplasm (ICD-10 codes C00-D48), this was considered as the primary tumour in the analysis. In cases where multiple reasons of death were reported, the first neoplasm diagnosis (ICD-10 codes C00-D48) among the reported codes was used.

The quality of the Swedish CoD register is described as high with a completeness of 97%. While the autopsy rates have dropped during in the last decades, the quality of the reporting was not necessarily negatively affected thanks to better diagnostic tools.[11] A retrospective study on 5675

For peer review only - http://bmjopen.bmj.com/site/about/guidelines.xhtml

patients with prostate cancer published in 2009 reported a 86% correlation between the registered CoD and the CoD according to the reviewed records.[12]

Indications for surgery

In Swespine, the surgeon reports the indication for surgery. The possible indications are neurologic deficits, pain, progressing deformity or any combination of those.

Neurologic function

The patient's neurologic function is reported according to the Frankel scale A-E, where A means a complete neurologic deficit and E means full motoric and sensory function.[13]

Types of surgery

The surgeon reports the type of decompressive surgery (posterior/anterior and levels) and types of implants used (yes/no to posterior and anterior implants) to Swespine. If both questions regarding implants were answered "no", we assumed that the patient was operated with decompression only.

In Swespine, the spinal levels of surgery are reported by the surgeon. The levels of decompression and stabilisation (if applicable) are reported, as well as the most proximal level of tumour mass addressed by the procedure. The most proximal level of tumour mass is used in this study to determine if the tumour mass is on cervical, thoracic or lumbosacral level. The decompression and/or stabilisation might cover more proximal levels than the reported level.

Quality of life

The patients are requested to assess their HRQoL before surgery by filling in an EQ-5D form. EQ-5D measures HRQoL in five dimensions and is widely used and validated in several medical fields.[14] Six weeks after surgery, the patient is requested to repeat the questionnaire and the values before and after surgery are reported to Swespine.

For peer review only

Data Sources / measurement

The spinal metastasis registry extract was provided by Swespine and sent to the Swedish National Board of Health and Welfare, where dates and causes of death were linked with the spinal metastasis registry dataset. An anonymised dataset was provided to the authors while the key remained with the agency.

Bias

All patients in this cohort were selected to undergo surgery and had probably better performance status than patients not selected for surgery. There a probable attrition bias in reporting QoL as patients with low performance status could be assumed to have less interest in filling out questionnaires, including the questionnaire six weeks post-operatively.

Registration of patient data in quality registers in Sweden is regulated by law and it is assumed that the patient agrees to participation, unless otherwise stated.[15] This reduces the risk of selection bias.

Study size

The study size was determined by the number of patients with spinal metastatic disease registered in Swespine. No power calculations were made prior to the study.

Quantitative variables

Type of surgery

The surgeon-reported data on surgery includes type of surgery (anterior and/or posterior decompression, type of implant used) and spinal levels of surgery. The most proximal part of tumour mass addressed by the surgery is registered and this value is used to classify the metastases as cervical, thoracic or lumbosacral.

For peer review only

Statistical methods

All calculations were made with the statistical software IBM SPSS Statistics version 26, including the survival estimations. The survival analysis was with the Kaplan Meier method. To test the statistical significance in survival between groups, the Breslow test was used.

Data access and cleaning methods

The authors have full access to the Swespine database, and the anonymised dataset with the patients in the spinal metastases cohort. This dataset matches the dataset received from the CoD register and no further cleaning of cases has been made.

Linkage

The authors were provided with an already linked dataset. The linkage was performed by the National Board of Health and Welfare.

Results

Participants

1820 adult patients were included in the analysis. The patients were treated at 19 different hospitals in Sweden. Six university hospitals accounted for 1646 (90%) of the cases.

Descriptive data

The included patients were 18 to 95 years old at the time of surgery (mean age 67 years, 67% male).

For peer review only

For peer review only - http://bmjopen.bmj.com/site/about/guidelines.xhtml

Outcome data

Survival

The estimated median survival after surgery was 6.2 months (95% CI 5.6-6.8) and the mean survival 12.2 months (95% CI 11.4-13.1). As of January 31st 2016, 531 (29.2%) of the patients were alive and 1289 (70.8%) were deceased. 49.0 percent were dead within six months after surgery and 68.6 percent were dead within twelve months after surgery. (Figure 2)

Cause of death

1253 of the deceased patients (97%) had a tumour diagnosis (ICD 10 codes C00-D48) listed as the main or contributing cause of death, according to the CoD register. In 26 of the cases (2.0%) no neoplasm diagnosis was reported as a contributing cause of death. Ten patients (0.7%) had R99.9 (Death NOS) as cause of death, indicating missing data.

Survival per tumour type

In 773 cases (45.8%), the tumour type was known before surgery according to Swespine, while it was stated as unknown in 301 cases (17.9%). There was missing data regarding 612 cases (36.3%). (Table 1)

The median estimated survival after surgery for patients with known primary tumour was 6.3 months and mean survival 12.0 months. For unknown primary tumour, median survival was estimated to 5.3 months and mean survival 14.6 months. This difference was not statistically significant (p=0.68). (Table 2)

Stratified estimated survival data for the three largest specified tumour groups (prostate, breast, lung) were calculated both from Swespine and the CoD register. In Swespine, a total of 429 patients had any of these tumour types registered. The CoD register, which includes 1289 patients from the

For peer review only - http://bmjopen.bmj.com/site/about/guidelines.xhtml

total cohort, had 679 patients with any of these diagnoses. There is an overlap between the registers, as cases might be registered correctly both in Swespine and the CoD register. On the other hand, patients still alive per December 31st 2016 are not included in the CoD register. Using the Breslow test, we found no significant differences in survival between the groups registered in Swespine and the groups with data from the CoD register, despite obvious sample size differences. Of the three sub-analysed tumour groups, patients with breast cancer had the longest estimated median and mean survival. (Table 3, Figure 3)

Indications for surgery

The most common indication for surgery was neurologic deficit, either alone or in combination with other symptoms. Data on indication was missing in 41%of the cases (Table 4).

Neurologic function

The neurologic status before surgery according to the Frankel scale is reported to Swespine. Frankel level C was the most commonly reported level while data was missing in 43 of the cases. (Table 5)

Types of surgery

Posterior decompression was performed in 1592 (87.4%) cases while 141 (7.7%) patients were decompressed anteriorly. 63 patients (3.5%) were decompressed both anteriorly and posteriorly. Data regarding decompression was missing in 1.3 percent of the cases. It is unclear from the data if this means that no decompression was done. (Table 6)

Types of implants

The most common surgical procedure in this material was decompression combined with stabilisation. Posterior implants were used alone or in combination with anterior implants in 70.5 percent of the cases. In 378 cases no implants were used at all. It is unclear whether data missing indicates that no implants were used or not. The type of implant (model/manufacturer) is reported

For peer review only - http://bmjopen.bmj.com/site/about/guidelines.xhtml

to Swespine and the most common type used in this cohort was a posterior pedicle screw system. (Table 7)

Spinal levels of surgery

Thoracic lesions were far more common than lumbosacral and cervical lesions. Th4 is the most common proximal level addressed by surgery in this material. (Figure 4)

Quality of life

640 of the patients (35.2%) had a registered value for EQ-5D as assessed prior to surgery and 909 patients (49.9%) had EQ-5D data registered six weeks after surgery. The concordance of the two groups was low, as only 411 (22.6%) had data registered both before and after surgery. This subgroup had a higher mean EQ-5D level before surgery as well as after surgery. (Table 8)

Discussion

Key results

This is one of the largest single-country cohorts with prospective baseline data, reliable survival data and fairly recently treated patients. The Swedish system with a unique PIN makes it possible to cross-reference and obtain data from several registries, which is one of the strengths with this study.

The median survival after surgery for metastatic spine disease in this cohort is by margin above the levels recommended in predictive scoring systems where there is a time-based level for expected survival (usually three months), indicating that more patients could be eligible for surgery given the relatively long survival.[16] The estimated mean survival is almost twice as long as the estimated median survival (12.2 months vs 6.2 months) which can be explained by the minority of patients living several years after the surgical treatment. As previously shown by our group, the use of old

For peer review only - http://bmjopen.bmj.com/site/about/guidelines.xhtml

prediction models may withhold patients from surgery as they tend to underestimate survival, and this study shows once again the need for reliable prediction tools.[17]

The Global Spine Tumour Study Group (GSTSG) has published several articles based on an international cohort of comparable size. While the patients in that cohort show higher survival rate one year after surgery, the mean age is lower (60 years versus 67 years in our material) and the distribution of cancer types is different. Patients with breast cancer and lung cancer were both more common than prostate cancer in the GSTSG cohort and sarcomas were included as well. The heterogeneity between these cohorts complicates any comparative analysis.[3, 18]

Not surprisingly, almost every patient with spinal metastatic disease in our cohort died of cancer rather than of other reasons. This emphasises the need to minimise complications after surgery in this fragile group.

Neurologic deficit was the most common indication for surgery reported in our material (38%), which is in line with the AOSpine Study published in 2015, where neurologic deficit was reported as the indication for surgery in 40% of the cases.[19] In our material, pain was the main indication for surgery in only nine percent of the cases while the AOSpine Study had “intractable pain” as main indication in 39% of the cases. Unfortunately, the rate of missing data regarding indication for surgery is high (43%) in our material which makes any conclusions uncertain. However, The AOSpine cohort is more recent than ours and we believe the difference illustrates the development in this field of spine surgery, where pain and instability are gaining more importance as indications. The Spine Instability Neoplastic Score (SINS), presented in 2010, has been validated as being correlated to patient-reported outcome after surgery which underlines the importance of pain and instability as indications for surgery. [20, 21]

The focus of this study is on survival after surgery, which is an important factor when making treatment decisions. However, we recognise that the main goal of the treatment is not to prolong the life but rather to increase the HRQoL for the patient. Unfortunately, the amount of missing data

For peer review only - http://bmjopen.bmj.com/site/about/guidelines.xhtml

regarding HRQoL in the spinal metastasis module of Swespine is very high. Almost eight out of ten patients have missing data on HRQoL and any conclusions from this material should be drawn with caution. As expected, the mean EQ-5D in the group being assessed both before and after surgery was higher than in the other groups only answering one of the questionnaires. This subgroup increased the mean EQ-5D score with 0.24, far above the suggested minimally important difference which is around 0.10 for cancer patients.[22]

Among those who assessed their HRQoL before surgery, the EQ-5D was low with a mean of 0.11. This level is comparable to the mean EQ-5D for dementia and lower than for palliative breast cancer and cerebral haemorrhage. [23]

The distribution of the metastases in the spine, where the thoracic spine accounts for the majority followed by the lumbar and cervical spines, is in line with other recent findings.[24, 25]

Limitations

The main limitation of this study is the lack of a non-surgical control group and the lack of reliable reporting of adverse events. Swespine has a system for reporting adverse advents in connection to the surgery, but finding late complications is a cumbersome task including manual assessment of multiple medical records and out of the scope for this study. Other authors have concluded that the rate of adverse events after spine surgery probably is under-reported and we have no reason to believe that the situation is different in Sweden.[26]

Several outcomes in Swespine have a high level of missing data, which is another obvious limitation. With a completeness of the register of around 75%, we can assume that there are several hundred cases during the period which are not entered into Swespine and thus not included in our study.

For peer review only

Interpretation

Survival after surgery for spinal metastatic disease in this cohort s well above the recommended minimum and surgery might be considered in even more cases. Further research should emphasise on prediction of survival and evaluation of HRQoL.

Generalisability

The results of this study do not apply to all patients with spinal metastatic disease, given the heterogeneity between different cancer forms and the lack of a non-surgical control group.

Footnotes

Author Contributions

CC drafted the manuscript and made the statistical analyses. CO was involved in the study design and reviewed the manuscript. YR designed the study, wrote the ethical board application and reviewed the manuscript.

Funding

This research received no specific grant from any funding agency in the public, commercial or not-for-profit sectors.

Competing interests

Dr. Carrwik and Dr. Robinson have nothing to disclose. Dr. Olerud reports personal fees from Johnson & Johnson (paid speaker on course) outside the submitted work.

For peer review only

Ethics approval

This study was approved by the Regional Ethical Review Board in Uppsala (no. 2012/133).

Data availability statement

Source data was accessed from Swespine and The National Cause of Death Register. Swespine is available to researchers subject to application http://www.swespine.se and the National Cause of Death Register is open-access, see https://www.socialstyrelsen.se/en/statistics-and-data/registers/

Tables

Type of primary tumour	n	%
Known	773	47.5
Unknown	301	18.5
Data missing	612	37.6
**Total**	**1626**	**100**

Table 1. Primary tumour as known before surgery reported to Swespine.

Primary tumour	Cases	Median estimated survival after surgery, months (95% CI)	Mean estimated survival after surgery, months (95% CI)
Known (n=773)	773	6.3 (5.4-7.2)	12.0 (10.9-13.2)
Unknown (n=301)	301	5.3 (3.8-6.8)	14.6 (12.0-17.2)

Table 2. Estimated survival after surgery for patients with known versus unknown primary tumour at the time of surgery, as reported to Swespine.

Primary tumour	Cases in the CoD register	Median estimated survival after surgery, months (95% CI)	Mean estimated survival after surgery, months (95% CI)	Cases in Swespine	Median estimated survival after surgery, months (95% CI)	Mean estimated survival after surgery, months (95% CI)	p-value
Prostate	405	7.2 (6.2-8.2)	11.5 (10.2-12.8)	277	7.6 (6.4-8.9)	12.6 (10.9-14.3)	0.25

For peer review only - http://bmjopen.bmj.com/site/about/guidelines.xhtml

Breast	111	10.4 (5.2-15.6)	17.7 (14.6-20.9)	74	8.5 (5.4-11.6)	17.7 (13.4-22.0)	0.46
Lung	163	3.4 (2.4-4.4)	7.6 (6.0-9.3)	78	3.2 (2.6-3.8)	6.8 (4.8-8.9)	0.71
Total	679			429			

1 Table 3. Survival per tumour type from the three largest groups, as reported in the CoD register and

2 Swespine respectively

**Indication(s) for surgery**	**n**	**%**	**Median estimated survival after surgery, months (95% CI)**	**Mean estimated survival after surgery, months (95% CI)**
Neurologic deficit (A)	684	38	5.8 (4.9-6.7)	12.6 (11.2-13.9)
Pain (B)	161	9	7.8 (5.8-9.8)	16.4 (12.8-19.9)
Progressive deformity (C)	19	1	6.5 (0.0-13.5)	15.8 (3.2-28.3)
A+B	154	9	5.3 (3.8-6.8)	10.2 (8.0-12.4)
A+C	11	0.6	2.8 (0.0-6.9)	6.0 (1.2-10.7)
B+C	20	1	5.3 (0.0-12.4)	9.2 (3.3-15.2)
A+B+C	31	2	11.6 (1.7-21.5)	17.1 (10.1-24.1)
Data missing	740	41		
**Total**	**1820**	**100**		

3 Table 4. Indications for surgery as reported to Swespine.

**Frankel level**	**n**	**%**	**Median estimated survival after surgery, months (95% CI)**	**Mean estimated survival after surgery, months (95% CI)**
A	37	2.0	4.0 (2.3-5.7)	9.4 (4.7-14.2)
B	78	4.3	5.7 1.9-9.5)	11.7 (7.6-15.6)
C	430	23.6	5.2 (4.0-6.4)	10.9 (9.4-12.4)
D	298	16.4	6.9 (5.0-8.8)	14.7 (11.7-15.7)
E	192	10.5	7.8 (5.0-10.6)	17.3 (13.8-20.9)
Data missing	785	43.1		
**Total**	**1820**	**100**		

4 Table 5. Neurologic status before surgery as reported to Swespine and estimated survival

For peer review only

For peer review only - http://bmjopen.bmj.com/site/about/guidelines.xhtml

Type of decompression	n	%	Median estimated survival after surgery, months (95% CI)	Mean estimated survival after surgery, months (95% CI)
Posterior	1592	87.5	5.4 (4.1-6.7)	12.0 (11.2-12.9)
Anterior	141	7.7	8.0 (4.0-12.0)	18.2 (13.4-23.1)
Posterior and anterior	63	3.5	9.4 (4.0-14.8)	21.1 (12.7-29.4)
Data missing	24	1.3		
**Total**	**1820**	**100**		

Table 6. Types of decompression as reported to Swespine and estimated survival.

Type(s) of implant	n	%	Median estimated survival after surgery, months (95% CI)	Mean estimated survival after surgery, months (95% CI)
Posterior only	1229	67.5	6.6 (5.8-7.4)	12.3 (11.3-13.3)
Anterior only	133	7.3	4.9 (1.4-8.4)	16.4 (11.7-12-0)
Posterior and anterior	55	3.0	11.4 (0.0-23.6)	23.5 (13.6-33.4)
No implant	378	20.7	4.1 (2.7-5.5)	12.3 (6.9-17.6)
Data missing	25	1.4		
**Total**	**1820**	**100**		

Table 7. Types of implants used as reported to Swespine and estimated survival.

EQ-5D assessment	n	%	EQ-5D mean	SD	Median estimated survival after surgery, months (95% CI)	Mean estimated survival after surgery, months (95% CI)
Before surgery	640	35.3	0.11	0.40	7.6 (6.6-8.6)	14.6 (13.0-16.2)
Six weeks after surgery	909	49.9	0.37	0.38	10.2 (9.0-11.4)	16.0 (14.8-17.3)
Subgroup A assessed both before and six weeks after surgery	411	22.6	-	-	11.4 (9.6-13.2)	17.6 (15.6-19-7)
Subgroup A: before surgery	411	100	0.14	0.39	-	-

For peer review only - http://bmjopen.bmj.com/site/about/guidelines.xhtml

Subgroup A: six weeks after surgery	411	100	0.38	0.39	-	-

Table 8. EQ-5D assessments and estimated survival in different subgroups, depending on data availability before and/or after surgery. Subgroup A had EQ-5D data both before and after surgery.

Figure Legends

Figure 1. Flowchart for inclusion and exclusion in the study.

Figure 2. Survival after surgery, all tumour types.

Figure 3. Survival after surgery per primary tumour.

Figure 4. Number of cases per proximal level addressed by surgery.

For peer review only

References

1. Patchell RA, Tibbs PA, Regine WF, Payne R, Saris S, Kryscio RJ, Mohiuddin M, Young B: **Direct decompressive surgical resection in the treatment of spinal cord compression caused by metastatic cancer: a randomised trial**. *The Lancet* 2005, **366**(9486):643-648.
2. Dea N, Versteeg A, Fisher C, Kelly A, Hartig D, Boyd M, Paquette S, Kwon BK, Dvorak M, Street J: **Adverse events in emergency oncological spine surgery: a prospective analysis**. *Journal of neurosurgery Spine* 2014:1-6.
3. Choi D, Ricciardi F, Arts M, Buchowski JM, Bunger C, Chung CK, Coppes M, Depreitere B, Fehlings M, Kawahara N *et al*: **Prediction Accuracy of Common Prognostic Scoring Systems for Metastatic Spine Disease: Results of a Prospective International Multicentre Study of 1469 Patients**. *Spine (Phila Pa 1976)* 2018, **43**(23):1678-1684.
4. Choi D, Fox Z, Albert T, Arts M, Balabaud L, Bunger C, Buchowski JM, Coppes MH, Depreitere B, Fehlings MG *et al*: **Prediction of Quality of Life and Survival After Surgery for Symptomatic Spinal Metastases: A Multicenter Cohort Study to Determine Suitability for Surgical Treatment**. *Neurosurgery* 2015, **77**(5):698-708; discussion 708.
5. Kim J, Lee S-H, Park S-J, Chung S-S, Kim E-S, Eoh W, Lee C-S: **Analysis of the predictive role and new proposal for surgical strategies based on the modified Tomita and Tokuhashi scoring systems for spinal metastasis**. *World journal of surgical oncology* 2014, **12**:245.
6. Torre LA, Siegel RL, Ward EM, Jemal A: **Global Cancer Incidence and Mortality Rates and Trends--An Update**. *Cancer epidemiology, biomarkers & prevention : a publication of the American Association for Cancer Research, cosponsored by the American Society of Preventive Oncology* 2016, **25**(1):16-27.
7. Wang M, Bünger CE, Li H, Wu C, Høy K, Niedermann B, Helmig P, Wang Y, Jensen AB, Schättiger K *et al*: **Predictive Value of Tokuhashi Scoring Systems in Spinal Metastases, Focusing on Various Primary Tumor Groups: Evaluation of 448 Patients in the Aarhus Spinal Metastases Database**. *Spine* 2012, **37**(7):573-582.
8. Benchimol EI, Smeeth L, Guttmann A, Harron K, Moher D, Petersen I, Sørensen HT, von Elm E, Langan SM, Committee RW: **The REporting of studies Conducted using Observational Routinely-collected health Data (RECORD) Statement**. *PLoS Med* 2015, **12**(10):e1001885.
9. Anell A, Glenngard AH, Merkur S: **Sweden health system review**. *Health systems in transition* 2012, **14**(5):1-159.
10. Stromqvist B, Fritzell P, Hagg O, Jonsson B, Sanden B: **Swespine: the Swedish spine register : the 2012 report**. *Eur Spine J* 2013, **22**(4):953-974.
11. Brooke HL, Talback M, Hornblad J, Johansson LA, Ludvigsson JF, Druid H, Feychting M, Ljung R: **The Swedish cause of death register**. *European journal of epidemiology* 2017, **32**(9):765-773.
12. Fall K, Stromberg F, Rosell J, Andren O, Varenhorst E: **Reliability of death certificates in prostate cancer patients**. *Scandinavian journal of urology and nephrology* 2008, **42**(4):352-357.
13. Frankel HL, Hancock DO, Hyslop G, Melzak J, Michaelis LS, Ungar GH, Vernon JD, Walsh JJ: **The value of postural reduction in the initial management of closed injuries of the spine with paraplegia and tetraplegia. I**. *Paraplegia* 1969, **7**(3):179-192.
14. Rabin R, de Charro F: **EQ-5D: a measure of health status from the EuroQol Group**. *Annals of medicine* 2001, **33**(5):337-343.
15. Levay C: **Policies to foster quality improvement registries: lessons from the Swedish case**. *Journal of internal medicine* 2016, **279**(2):160-172.

For peer review only - http://bmjopen.bmj.com/site/about/guidelines.xhtml

16. Tokuhashi Y, Matsuzaki H, Oda H, Oshima M, Ryu J: **A revised scoring system for preoperative evaluation of metastatic spine tumor prognosis**. *Spine (Phila Pa 1976)* 2005, **30**(19):2186-2191.
17. Carrwik C, Olerud C, Robinson Y: **Predictive Scores Underestimate Survival of Patients with Metastatic Spine Disease: A Retrospective Study of 315 Patients in Sweden**. *Spine (Phila Pa 1976)* 2019.
18. Depreitere B, Ricciardi F, Arts M, Balabaud L, Bunger C, Buchowski JM, Chung CK, Coppes MH, Fehlings MG, Kawahara N *et al*: **How good are the outcomes of instrumented debulking operations for symptomatic spinal metastases and how long do they stand? A subgroup analysis in the global spine tumor study group database**. *Acta neurochirurgica* 2020.
19. Fehlings MG, Nater A, Tetreault L, Kopjar B, Arnold P, Dekutoski M, Finkelstein J, Fisher C, France J, Gokaslan Z *et al*: **Survival and Clinical Outcomes in Surgically Treated Patients With Metastatic Epidural Spinal Cord Compression: Results of the Prospective Multicenter AOSpine Study**. *J Clin Oncol* 2015.
20. Fisher CG, DiPaola CP, Ryken TC, Bilsky MH, Shaffrey CI, Berven SH, Harrop JS, Fehlings MG, Boriani S, Chou D *et al*: **A novel classification system for spinal instability in neoplastic disease: an evidence-based approach and expert consensus from the Spine Oncology Study Group**. *Spine (Phila Pa 1976)* 2010, **35**(22):E1221-1229.
21. Versteeg AL, Sahgal A, Laufer I, Rhines LD, Sciubba DM, Schuster JM, Weber MH, Lazary A, Boriani S, Bettegowda C *et al*: **Correlation Between the Spinal Instability Neoplastic Score (SINS) and Patient Reported Outcomes**. *Global Spine Journal* 2021:219256822110335.
22. Pickard AS, Neary MP, Cella D: **Estimation of minimally important differences in EQ-5D utility and VAS scores in cancer**. *Health Qual Life Outcomes* 2007, **5**:70.
23. Van Wilder L, Rammant E, Clays E, Devleesschauwer B, Pauwels N, De Smedt D: **A comprehensive catalogue of EQ-5D scores in chronic disease: results of a systematic review**. *Quality of Life Research* 2019, **28**(12):3153-3161.
24. Amelot A, Terrier LM, Cristini J, LeNail LR, Buffenoir K, Pascal-Moussellard H, Bonaccorsi R, Mathon B: **Approaching spinal metastases spread profile**. *Surgical oncology* 2019, **31**:61-66.
25. Onken JS, Fekonja LS, Wehowsky R, Hubertus V, Vajkoczy P: **Metastatic dissemination patterns of different primary tumors to the spine and other bones**. *Clinical & experimental metastasis* 2019, **36**(6):493-498.
26. Dea N, Versteeg A, Fisher C, Kelly A, Hartig D, Boyd M, Paquette S, Kwon BK, Dvorak M, Street J: **Adverse events in emergency oncological spine surgery: a prospective analysis**. *Journal of neurosurgery Spine* 2014, **21**(5):698-703.

For peer review only

For peer review only - http://bmjopen.bmj.com/site/about/guidelines.xhtml

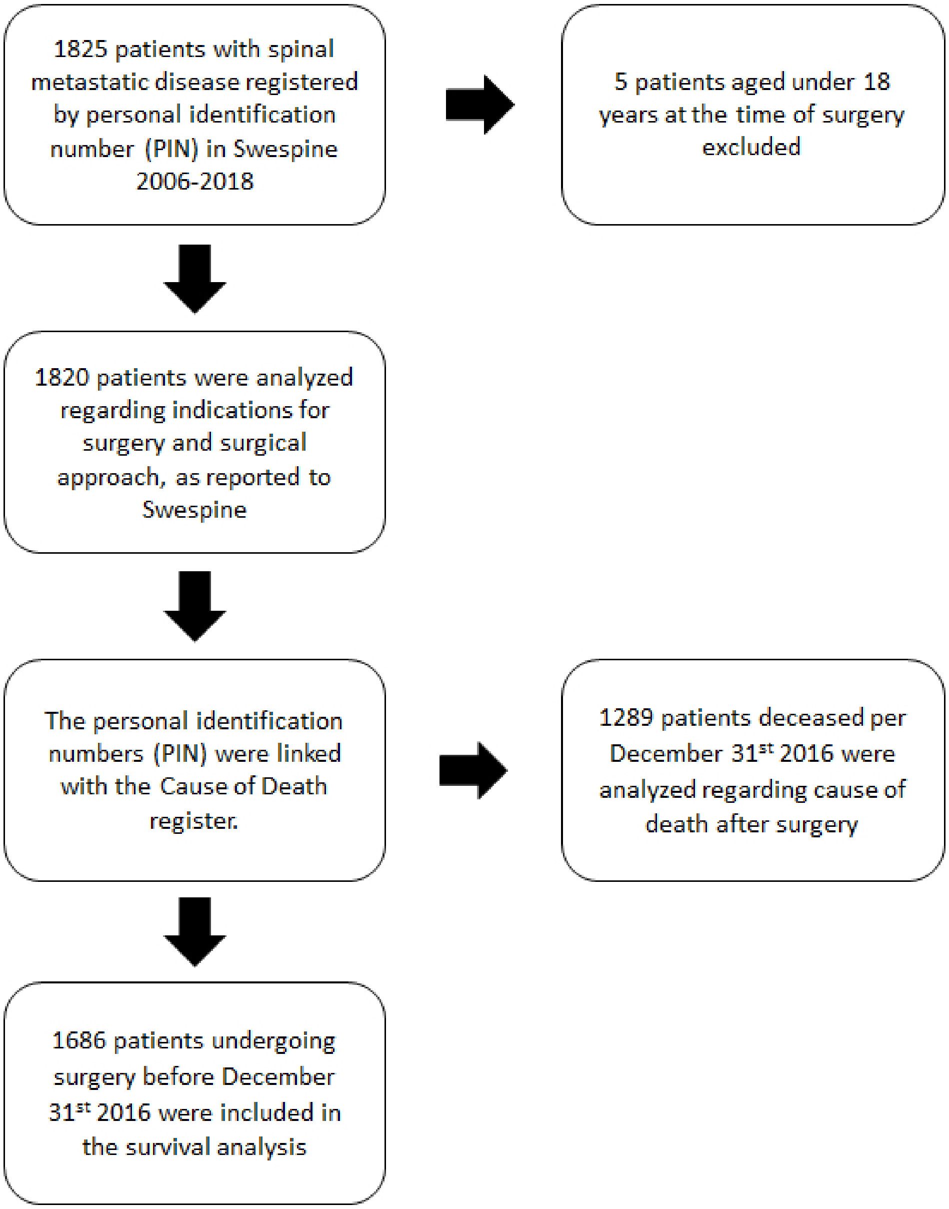

Flowchart for inclusion and exclusion in the study.

116x147mm (120 x 120 DPI)

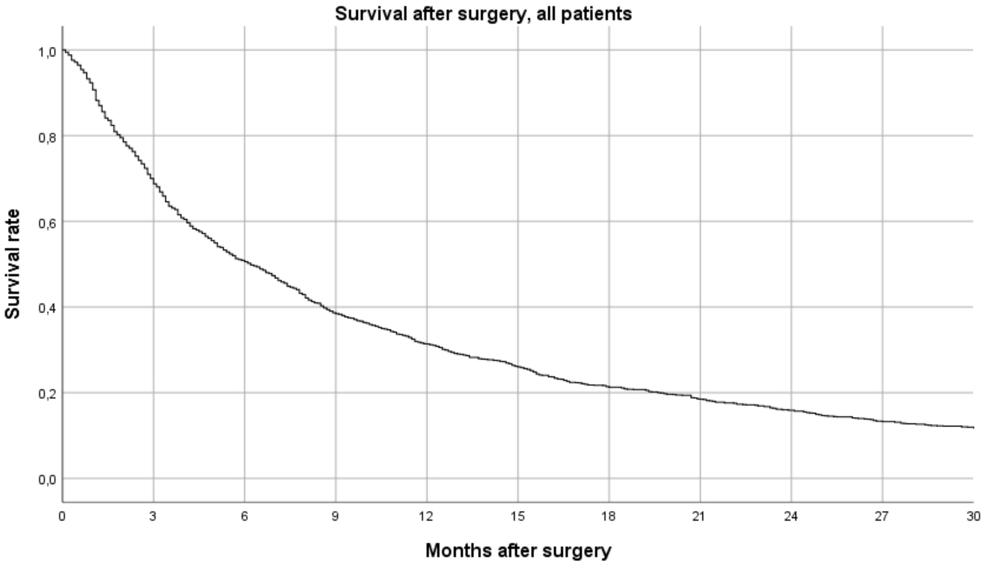

Survival after surgery, all tumour types.

179x107mm (120 x 120 DPI)

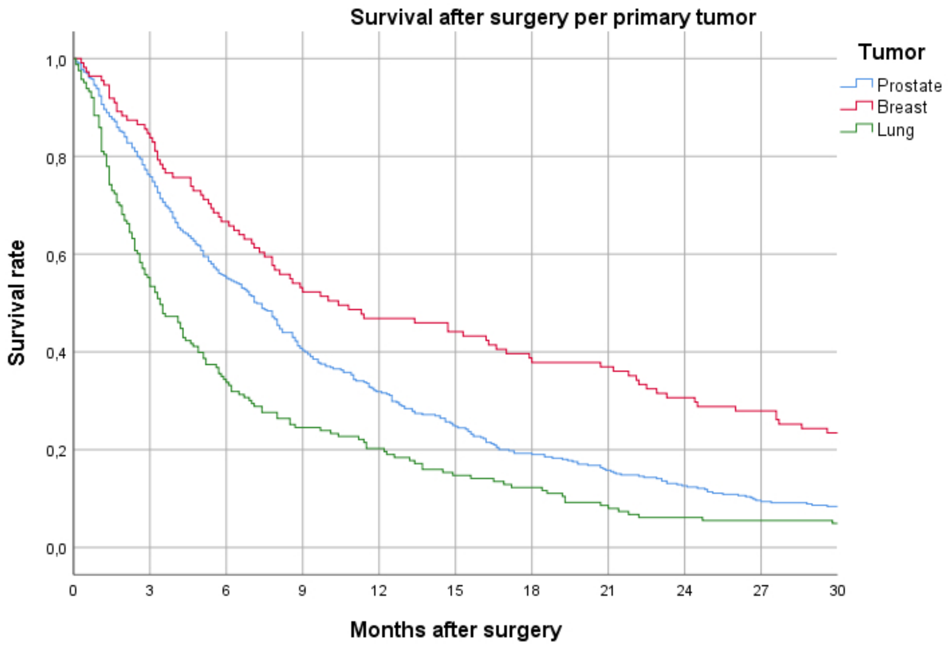

Survival after surgery per primary tumour.

157x108mm (120 x 120 DPI)

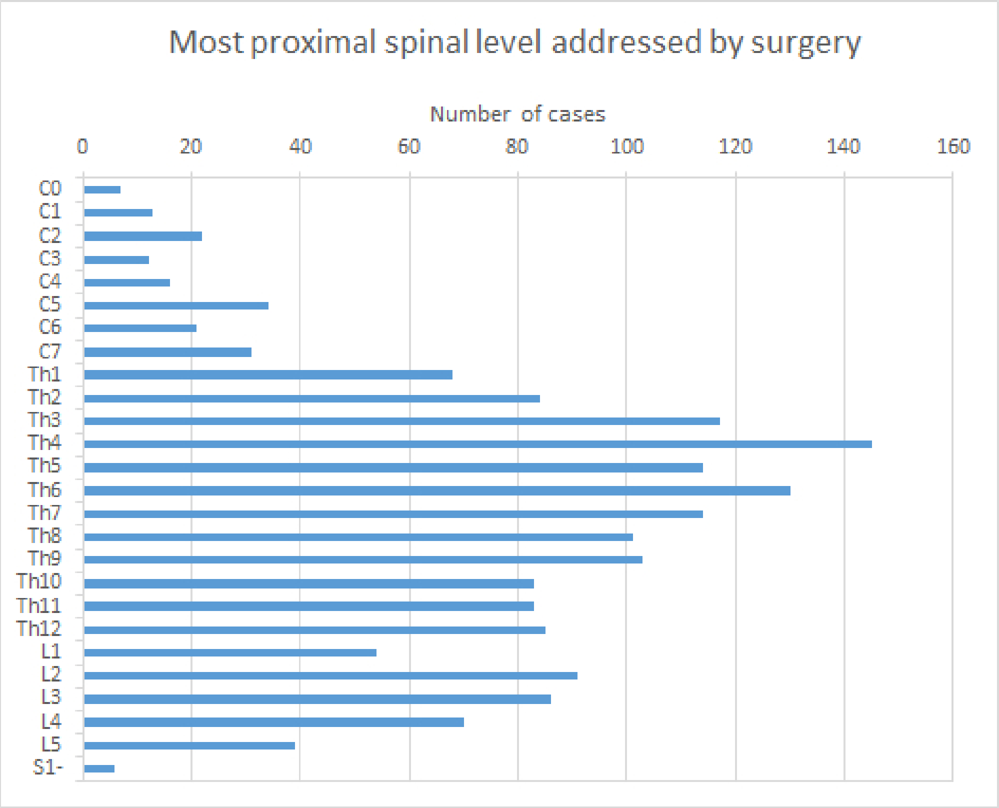

Number of cases per proximal level addressed by surgery.

111x90mm (120 x 120 DPI)

Reporting checklist for cohort study.

Based on the STROBE cohort guidelines.

Instructions to authors

Complete this checklist by entering the page numbers from your manuscript where readers will find each of the items listed below.

Your article may not currently address all the items on the checklist. Please modify your text to include the missing information. If you are certain that an item does not apply, please write "n/a" and provide a short explanation.

Upload your completed checklist as an extra file when you submit to a journal.

In your methods section, say that you used the STROBE cohortreporting guidelines, and cite them as:

von Elm E, Altman DG, Egger M, Pocock SJ, Gotzsche PC, Vandenbroucke JP. The Strengthening the Reporting of Observational Studies in Epidemiology (STROBE) Statement: guidelines for reporting observational studies.

		Reporting Item	Page Number
**Title and abstract**			
Title	#1a	Indicate the study’s design with a commonly used term in the title or the abstract	1

For peer review only

Abstract	#1b	Provide in the abstract an informative and balanced summary of what was done and what was found	2
**Introduction**			
Background / rationale	#2	Explain the scientific background and rationale for the investigation being reported	3
Objectives	#3	State specific objectives, including any prespecified hypotheses	2
**Methods**			
Study design	#4	Present key elements of study design early in the paper	2
Setting	#5	Describe the setting, locations, and relevant dates, including periods of recruitment, exposure, follow-up, and data collection	2
Eligibility criteria	#6a	Give the eligibility criteria, and the sources and methods of selection of participants. Describe methods of follow-up.	2
Eligibility criteria	#6b	For matched studies, give matching criteria and number of exposed and unexposed	5
Variables	#7	Clearly define all outcomes, exposures, predictors, potential confounders, and effect modifiers. Give diagnostic criteria, if applicable	5
Data sources / measurement	#8	For each variable of interest give sources of data and details of methods of assessment (measurement). Describe comparability of assessment methods if there is more than	7

For peer review only

		one group. Give information separately for for exposed and unexposed groups if applicable.	
Bias	#9	Describe any efforts to address potential sources of bias	7
Study size	#10	Explain how the study size was arrived at	7
Quantitative variables	#11	Explain how quantitative variables were handled in the analyses. If applicable, describe which groupings were chosen, and why	7
Statistical methods	#12a	Describe all statistical methods, including those used to control for confounding	
7			
Statistical methods	#12b	Describe any methods used to examine subgroups and interactions	7
Statistical methods	#12c	Explain how missing data were addressed	7
Statistical methods	#12d	If applicable, explain how loss to follow-up was addressed	7
Statistical methods	#12e	Describe any sensitivity analyses	
7			
**Results**			
Participants	#13a	Report numbers of individuals at each stage of study—eg numbers potentially eligible, examined for eligibility,	8

For peer review only

		confirmed eligible, included in the study, completing follow-up, and analysed. Give information separately for for exposed and unexposed groups if applicable.	
Participants	#13b	Give reasons for non-participation at each stage	8
Participants	#13c	Consider use of a flow diagram	
8			
Descriptive data	#14a	Give characteristics of study participants (eg demographic, clinical, social) and information on exposures and potential confounders. Give information separately for exposed and unexposed groups if applicable.	8
Descriptive data	#14b	Indicate number of participants with missing data for each variable of interest	
8			
Descriptive data	#14c	Summarise follow-up time (eg, average and total amount)	
9			
Outcome data	#15	Report numbers of outcome events or summary measures over time. Give information separately for exposed and unexposed groups if applicable.	
9			
Main results	#16a	Give unadjusted estimates and, if applicable, confounder-adjusted estimates and their precision (eg, 95% confidence	8

For peer review only

		interval). Make clear which confounders were adjusted for and why they were included	
Main results	#16b	Report category boundaries when continuous variables were categorized	8
Main results	#16c	If relevant, consider translating estimates of relative risk into absolute risk for a meaningful time period	
8			
Other analyses	#17	Report other analyses done—eg analyses of subgroups and interactions, and sensitivity analyses	8
**Discussion**			
Key results	#18	Summarise key results with reference to study objectives	11
Limitations	#19	Discuss limitations of the study, taking into account sources of potential bias or imprecision. Discuss both direction and magnitude of any potential bias.	11
Interpretation	#20	Give a cautious overall interpretation considering objectives, limitations, multiplicity of analyses, results from similar studies, and other relevant evidence.	12
Generalisability	#21	Discuss the generalisability (external validity) of the study results	13
**Other Information**			

For peer review only

Funding	#22	Give the source of funding and the role of the funders for the present study and, if applicable, for the original study on which the present article is based	13

The STROBE checklist is distributed under the terms of the Creative Commons Attribution License CC-BY. This checklist was completed on 18. January 2021 using https://www.goodreports.org/, a tool made by the EQUATOR Network in collaboration with Penelope.ai

For peer review only
